# A Split-GFP Gateway Cloning System for Topology Analyses of Membrane Proteins in Plants

**DOI:** 10.1371/journal.pone.0170118

**Published:** 2017-01-13

**Authors:** Wenjun Xie, Mads Eggert Nielsen, Carsten Pedersen, Hans Thordal-Christensen

**Affiliations:** Plant Defence Genetics, Department of Plant and Environmental Sciences, University of Copenhagen, Thorvaldsensvej 40, Frederiksberg C, Denmark; Universiteit Gent, BELGIUM

## Abstract

To understand the function of membrane proteins, it is imperative to know their topology. For such studies, a split green fluorescent protein (GFP) method is useful. GFP is barrel-shaped, consisting of 11 β-sheets. When the first ten β-sheets (GFP1-10) and the 11th β-sheet (GFP11) are expressed from separate genes they will self-assembly and reconstitute a fluorescent GFP protein. However, this will only occur when the two domains co-localize in the same cellular compartment. We have developed an easy-to-use Gateway vector set for determining on which side of the membrane the N- and C-termini are located. Two vectors were designed for making N- and C-terminal fusions between the membrane proteins-of-interest and GFP11, while another three plasmids were designed to express GFP1-10 in either the cytosol, the endoplasmic reticulum (ER) lumen or the apoplast. We tested functionality of the system by applying the vector set for the transmembrane domain, CNX^TM^, of the ER membrane protein, calnexin, after transient expression in *Nicotiana benthamiana* leaves. We observed GFP signal from the ER when we reciprocally co-expressed GFP11-CNX^TM^ with GFP1-10-HDEL and CNX^TM^-GFP with cytosolic GFP1-10. The opposite combinations did not result in GFP signal emission. This test using the calnexin ER-membrane domain demonstrated its C-terminus to be in the cytosol and its N-terminus in the ER lumen. This result confirmed the known topology of calnexin, and we therefore consider this split-GFP system highly useful for ER membrane topology studies. Furthermore, the vector set provided is useful for detecting the topology of proteins on other membranes in the cell, which we confirmed for a plasma membrane syntaxin. The set of five Ti-plasmids are easily and efficiently used for Gateway cloning and transient transformation of *N*. *benthamiana* leaves.

## Introduction

Novel membrane proteins are still being identified in for instance the endoplasmic reticulum (ER) and the plasma membrane (PM) [1–4], and insight in their topology is essential for unraveling their function [1,5]. Several experimental and software-based prediction methods are available for ER as well as other membrane proteins [6–11]. A number of these experimental methods make use of fusing a fluorescent protein to the membrane protein-of-interest. Gross et al. [6] used reconstitution of split green fluorescent protein (GFP) for topology studies of chloroplastic, mitochondrial and peroxisomal membrane proteins in plant protoplasts. In the present study, we developed an easy-to-use method also using reconstitution of split-GFP for topology studies of membrane proteins in intact plants, as previously described for e.g. ER proteins in a rabbit kidney cell line [7]. These studies benefit from the work of Cabantous et al. [12] and Cabantous and Walde [13], who developed a mutant version of GFP that allowed the first ten β-sheets (GFP1-10) and the eleventh β-sheet (GFP11) to self-assemble and fluoresce when expressed separately.

Here we developed a set of plasmids to facilitate plant membrane protein topology studies. Three plasmids encode soluble GFP1-10 to be expressed either in the cytosol, in the ER lumen or in the apoplast. Another two plasmids were generated as Gateway destination vectors to make N- and C-terminal GFP11 fusions with proteins-of-interest. To subsequently test this membrane protein topology analysis system, we selected to use the transmembrane domain (CNX^TM^) of the *Arabidopsis* ER protein, calnexin 1. Calnexins are ER-membrane integral proteins with an N-terminal Ca^2+^-binding chaperone domain in the ER lumen and a C-terminal transmembrane domain [14,15]. Only when CNX^TM^-GFP11 was co-expressed with cytosolic GFP1-10 and when GFP11-CNX^TM^ was co-expressed with ER-luminal GFP1-10, did we observe strong GFP signal at the ER cortical network in *Nicotiana benthamiana* leaf epidermal cells. In the other two combinations, no signal was observed. Similarly, the system was tested using the plasma membrane syntaxin, PEN1 (SYP121) [16,17], and overall our results illustrate the usefulness of this split-GFP system for membrane protein topology analyses in plants.

## Materials and Methods

### Construct design

A DNA sequence, consisting of a sequence encoding the signal peptide (SP) of the *Arabidopsis* PR-1 gene (At2g14610), a sequence encoding the modified GFP1-10 [13] and a coding sequence of the HDEL ER retention signal, all flanked by the Gateway attB1/attB2 recombination cassette, was synthesized by Genscript, NJ, USA. The GFP1-10-HDEL coding sequence was, via recombination into pDONR221 (Invitrogen), subsequently recombined into the T-DNA destination vector, pB2GW7 [18], for CaMV35S-driven transcription. This generated the pSP-GFP1-10-HDEL Ti-plasmid for expression of ER-luminal GFP1-10 (S1A Fig).

Four restriction sites had been introduced in the SP-GFP1-10-HDEL coding sequence. These were two *Xho*I sites on each side of the ATG start codon and two *Hin*dIII sites on each side of the HDEL coding sequence. After digesting the SP-GFP1-10-HDEL insert when present in pDONR221 either with *Xho*I and *Hin*dIII in combination or with *Hin*dIII alone, followed by re-ligation, two constructs were obtained. The first encoded only the original GFP1-10 including its ATG start codon, without signal peptide and C-terminal HDEL retention signal. The second construct encoded GFP1-10 with the signal peptide at its N-terminus, but without the HDEL. Recombining these into pB2GW7 using LR clonase reactions generated the pGFP1-10 and pSP-GFP1-10 Ti-plasmids for expression of cytosolic and secreted GFP1-10, respectively (S1B and S1C Fig).

Gateway destination vectors for GFP11-protein-of-interest fusions were generated by PCR using pB2GW7, with a protein coding sequence inserted, as template. For the N-terminal GFP11 fusion vector, a combinatorial forward primer (GFP11-M3-3F), consisting of the 3’ part of the CaMV35S promoter, the GFP11 M3 [12] coding sequence, including a start codon, and the 5’ part of attB1, was designed (S1 Table). A reverse primer (P35SR2) was designed from the CaMV35S terminator sequence (S1 Table). The amplification product, generated using Phusion® High-Fidelity DNA Polymerase (NEB), was purified and used as megaprimer for amplifying a Ti-plasmid encoding a GFP11 fusion version of the protein in 25 cycles. This plasmid was transformed into *Escherichia coli* after adding the restriction enzyme *Dpn*I to digest the original template plasmid. A single clone of the plasmid was selected. The Gateway cassette was amplified from pDONR221 using the M13F/M13R primer set, and recombined into the selected plasmid using a BP clonase reaction in order to make the final pGFP11-GW destination T-DNA vector (S1D Fig).

In a similar way, we made pGW-GFP11. Here we used a combinatorial reverse primer (GFP11-M3-4R) designed from the 5’ part of the CaMV35S terminator, the GFP11 coding sequence, with a stop codon, attB2 as well as four codons of the inserted coding sequence without stop codon (S1 Table). A forward primer (P35SF) was designed from the CaMV35S promoter (S1 Table). By using the same procedure as above, we made the final pGW-GFP11 destination T-DNA vector (S1E Fig).

The calnexin 1 transmembrane domain (CNX^TM^)-coding sequence with start codon, and with and without stop codon, was PCR amplified from *Arabidopsis* Col-0 genomic DNA using gene specific primers (S1 Table). The two amplified products were TOPO-cloned into pENTR-D (Invitrogen) and transferred to pK7WGF2 [18], pGW-GFP11, pGFP11-GW using LR clonase reactions. This generated the pGFP-CNX^TM^, pGFP11-CNX^TM^ and pCNX^TM^-GFP11 Ti-plasmids.

The PEN1-coding sequence was PCR-amplified from the GFP-PEN1 coding sequence described by Collins et al. [16] using gene specific primers with attB1 and attB2 adapter sites (S1 Table). The amplified product was re-amplified using attB1 and attB2 primers (S1 Table) and cloned into pDONR221 using a BP clonase reaction. To generate a PEN1 coding sequence without stop codon, we performed site-directed mutagenesis by PCR with the PEN1mutSTOP-F and PEN1mutSTOP-R primers, Phusion® and *Dpn*1 as described above to convert the stop codon (TGA) to encode Leu (TTA). The two PEN1 constructs were transferred to pGFP11-GW and pGW-GFP11 using LR clonase reactions. This generated the pGFP11-PEN1 and pPEN1-GFP11 Ti-plasmids. All plasmids were produced in *E*. *coli*, TOP10 and MACH1, and transformed into *Agrobacterium tumefaciens*, GV3101, for agroinfiltration.

All generated plasmids were confirmed by sequencing. The CLC software was used for sequence management and for plasmid map design.

The plasmids generated in this work are available upon request.

### Agroinfiltration and microscopy

*N*. *benthamiana* leaf agroinfiltration and confocal microscopy was performed as previously described [19]. Specifically, a Leica SP5 confocal laser scanning microscope was used. For detection and localization of the fluorophores, GFP was excited at 488 nm and detected between 500 and 530 nm, while RFP was excited at 543 nm and detected between 579 nm and 640 nm.

## Results and Discussion

**Fig 1:**
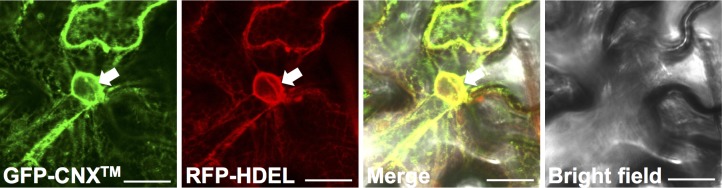
The transmembrane domain of calnexin 1, CNX^TM^, localizes to the ER membrane. Constructs encoding GFP-CNX^TM^ and the ER-luminal SP-RFP-HDEL were co-transformed into cells of *Nicotiana benthamiana* leaves by agroinfiltration. Confocal microscopy was performed 2 days after infiltration. Scale bar, 20 μm. Arrow, nucleus.

To initially confirm the ER localization of CNX^TM^, we transiently co-transformed *N*. *benthamiana* leaves with the GFP-CNX^TM^-expression construct and a construct expressing an ER-luminal marker, SP-RFP-HDEL [20], using agroinfiltration. Confocal microscopic analysis of the epidermal cells 2 days after bacterial infiltration showed overlap between the GFP and the RFP signals. Both signals were cortical network-like as well as perinuclear, but not intranuclear (Fig 1). This signal pattern is indicative of GFP and RFP being at the ER, agreeing with the ER membrane location of CNX^TM^ and the ER-luminal location of RFP-HDEL.

**Fig 2:**
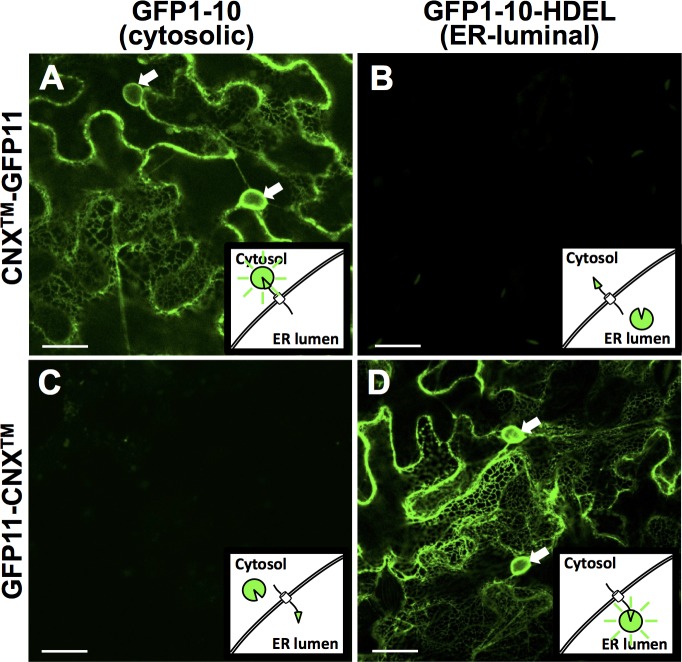
The transmembrane domain of calnexin 1 has its C-terminus in the cytosol and its N-terminus in the ER lumen. (A-D) Constructs encoding SP-GFP1-10-HDEL, GFP1-10, GFP11-CNX^TM^ and CNX^TM^-GFP11 were co-transformed pairwise into cells of *Nicotiana benthamiana* leaves by agroinfiltration. Confocal microscopy was performed 2 days after infiltration. Scale bars, 20 μm. Arrows, nuclei.

Next, the CNX^TM^ coding sequence, with and without stop codon, was inserted into the T-DNA Gateway destination vectors, pGFP11-GW and pGW-GFP11, to fuse GFP11 to the N- and C-terminus of CNX^TM^, respectively. The resulting constructs were co-transformed with constructs expressing either cytosolic GFP1-10 or ER-luminal SP-GFP1-10-HDEL into *N*. *benthamiana* leaves by agroinfiltration. Two days later, the leaf epidermal cells expressing all four construct-combinations were analyzed by confocal microscopy. Co-expression of GFP11, fused to the C-terminus of CNX^TM^, with cytosolic GFP1-10, resulted in strong GFP fluorescence at the ER cortical network. Together with a strong perinuclear signal and lack of intranuclear signal (Fig 2A), this confirmed the ER localization of CNX^TM^. The same signal pattern was observed after co-expression of GFP11, fused to the N-terminus of CNX^TM^, with ER-luminal GFP1-10 (Fig 2D). In the other two combinations, no signals were observed (Fig 2B and 2C). This showed that GFP11, fused to the cytosolic terminal of an ER membrane protein, was able to self-assemble with free cytosolic GFP1-10 to generate a fluorescent signal. The same was true when these two moieties were co-expressed on the luminal side of the ER membrane, despite the different redox and ionic conditions in this compartment. This was in agreement with previous use of split-GFP in mammalian cells and the known topology of calnexin [7,15].

**Fig 3:**
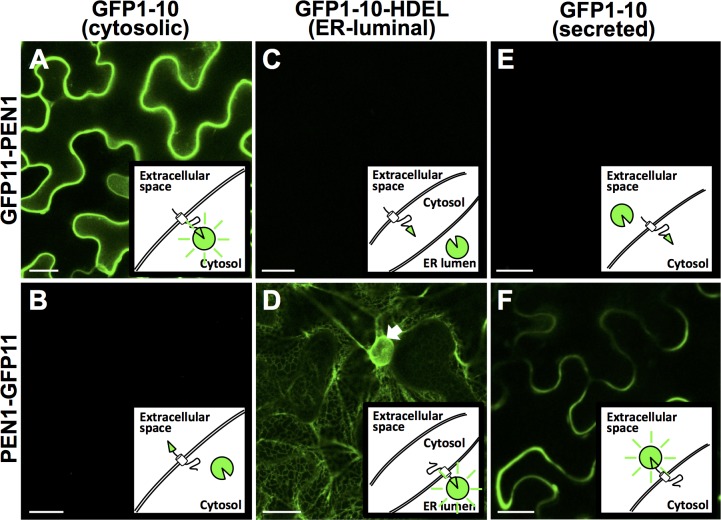
The plasma membrane syntaxin, PEN1, has its N-terminus in the cytosol and C-terminal in the apoplast. (A-F) Constructs encoding GFP1-10, SP-GFP1-10-HDEL, SP-GFP1-10, GFP11-PEN1 and PEN1-GFP11 were co-transformed pairwise into cells of *Nicotiana benthamiana* leaves by agroinfiltration. Confocal microscopy was performed 2 days after infiltration. Scale bars, 20 μm. Arrow, nucleus.

To explore the usefulness of the method for topology analyses of proteins integral to other membranes, we selected to test the plasma membrane (PM) syntaxin, PEN1 (SYP121) [16,17]. Syntaxins have a single transmembrane domain, their N-terminus in the cytosol and their C-terminus in the lumen of the membrane system or outside the cell, depending on which membrane they are embedded in. As expected, strong GFP fluorescence was observed at the PM after co-expression of PEN1, which has GFP11 fused to its N-terminus, and cytosolic GFP1-10 (Fig 3A). When GFP11 was fused to the C-terminus of PEN1, no signal was obtained with cytosolic GFP1-10 (Fig 3B). This agreed with the known topology of syntaxins, and showed that the split-GFP system also functions for proteins at other membranes, as previously noted by Gross et al. [6]. However, when the PEN1-GFP11 construct was co-expressed with the ER-luminal GFP1-10, we observed that the split-GFP-system cannot be used uncritically for protein localization. In this case we observed GFP signal from the ER (Fig 3D), and we consider this to be caused by the HDEL ER-retention sequences on GFP1-10. When the PEN1-C-terminally-linked GFP11 β-sheet self-assembled with the ER-retained GFP1-10, we believe PEN1 was returned to the ER from the *cis*-Golgi by retrograde membrane traffic, resulting in the clear ER-GFP signal. This observation indicates that the GFP11 β-sheet binds to GFP1-10 with a significant strength, and it further supports that the split-GFP-system is useful for membrane topology studies. However, it also provides the important note that the split-GFP system cannot be used for protein localization *per se*. To overcome this problem of mis-localization, we co-expressed the PEN1-GFP11 construct with a construct for (secreted) SP-GFP1-10. This resulted in GFP signal at the PM (Fig 3F) like previously observed when GFP11-PEN1 was combined with cytosolic GFP1-10 (Fig 3A). Notably, the PEN1-GFP11/secreted GFP1-10-combination caused a reduced signal, likely due to the lower extracellular pH known to affect the fluorescence of GFP [21]. Otherwise, when GFP11-PEN1 was co-expressed with ER-luminal or secreted GFP1-10 (Fig 3C and 3E), no GFP signal was detected, reflecting that the two GFP moieties never co-exist in a compartment.

In conclusion, this Gateway system offers an easy-to-use, fast and robust method, useful for uncovering the topology of novel plant proteins found to be membrane-associated. When, as in the case of CNX, a protein has its termini on each side of the membrane, then the method is self-confirmatory in its reciprocality. Meanwhile, it will occur in topology studies that GFP1-10 constructs do not reconstitute with a counterpart to result in fluorescence. To confirm the expression and functionality of such constructs, we recommend combining them with for instance our CNX^TM^/GFP11 constructs to establish positive controls. A scenario where this would be required is when a membrane protein has its N- and C-termini on the same side of the membrane. Otherwise, it is worth mentioning that GFP11, to be fused onto the protein-of-interest, consists of only 16 amino acids. With this small size, there is minimal risk that it inflicts artefacts influencing the result. An exception will be when the interacting GFP1-10 is limited in its mobility.

## Supporting Information

S1 Fig Map of plasmids made in this work. A) pSP-GFP1-10-HDEL, B) pGFP1-10, C) pSP-GFP1-10, D) pGFP11-GW, E) pGW-GFP11.
(PDF)

S1 Table Primers used in this work. (PDF)
