# Supplementary material for: A Split-GFP Gateway Cloning System for Topology Analyses of Membrane Proteins in Plants
Source: PLoS One. 2017 Jan 13;12(1):e0170118. doi: 10.1371/journal.pone.0170118 (PMC5234810; doi:10.1371/journal.pone.0170118)
Supplement: S1 Fig — A) pSP-GFP1-10-HDEL, B) pGFP1-10, C) pSP-GFP1-10, D) pGFP11-GW, E) pGW-GFP11. (PDF) [file pone.0170118.s001.pdf]

A Split-GFP Gateway Cloning System for Topology Analyses of Membrane Proteins in Plants

Wenjun Xie, Mads Eggert Nielsen, Carsten Pedersen and Hans Thordal-Christensen

Plant Defence Genetics, Department of Plant and Environmental Sciences, University of Copenhagen, Thorvaldsensvej 40, DK-1871 Frederiksberg C, Denmark

S1 Fig. Map of plasmids made in this work.

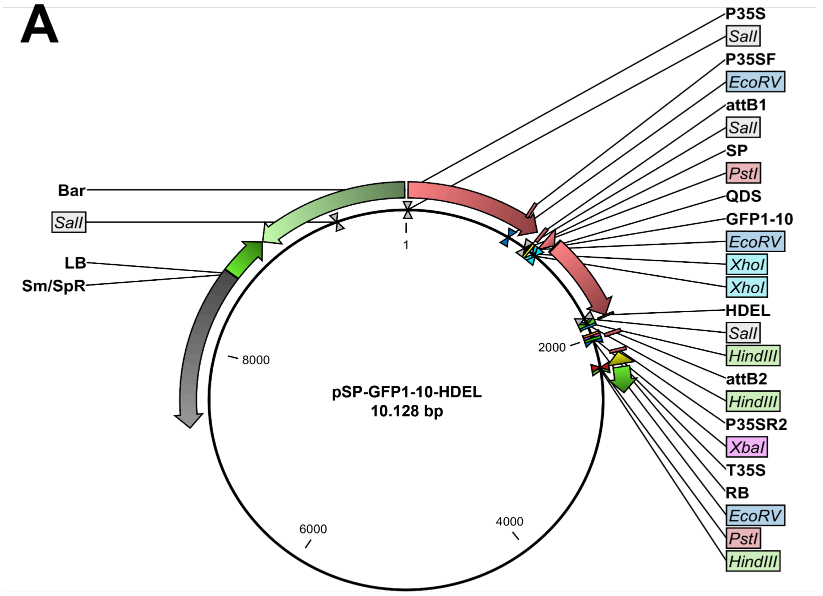

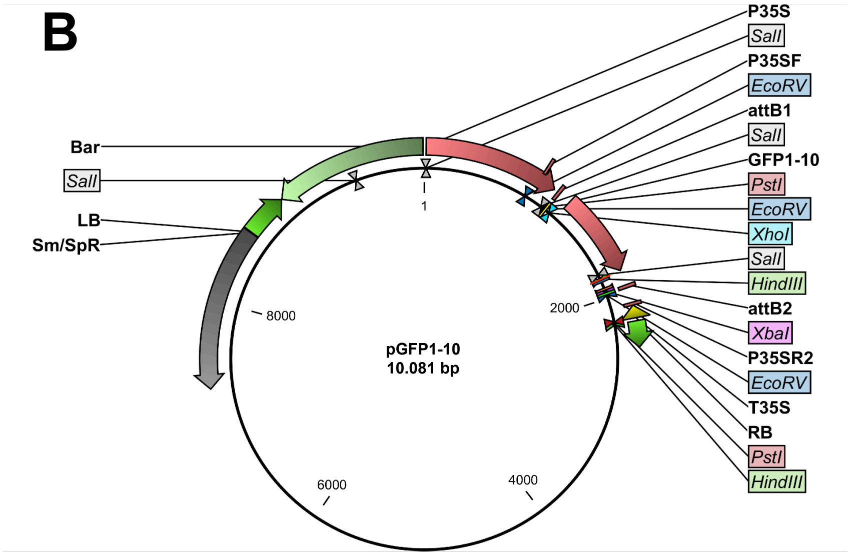

C

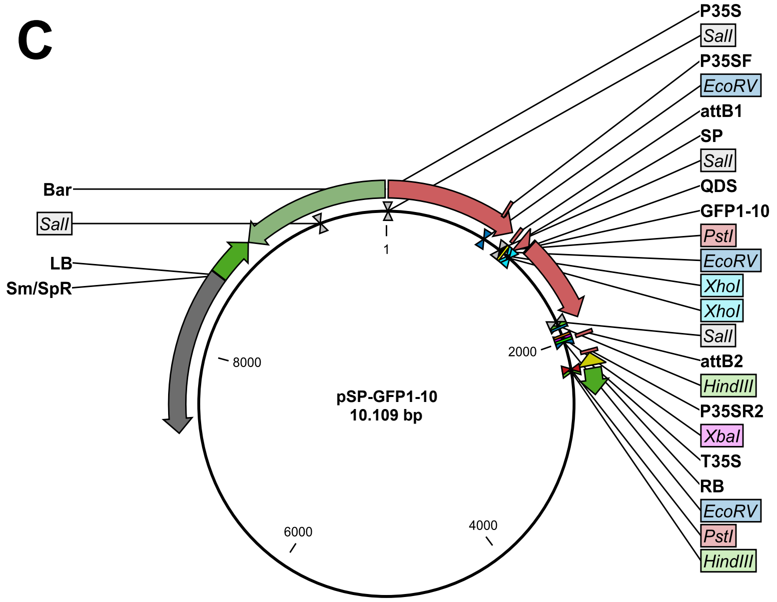
pSP-GFP1-10
10.109 bp
1
2000
4000
6000
8000
Bar
SalI
LB
Sm/SpR
P35S
SalI
P35SF
EcoRV
attB1
SP
SalI
QDS
GFP1-10
PstI
EcoRV
XhoI
XhoI
SalI
attB2
HindIII
P35SR2
XbaI
T35S
RB
EcoRV
PstI
HindIII

D

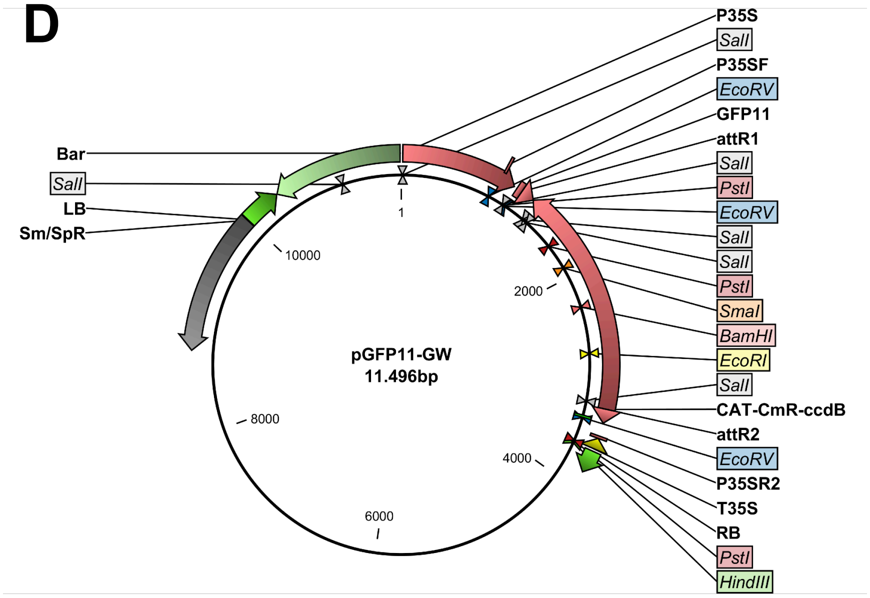
pGFP11-GW
11.496bp
1
2000
4000
6000
8000
10000
Bar
SalI
LB
Sm/SpR
P35S
SalI
P35SF
EcoRV
GFP11
attR1
SalI
PstI
EcoRV
SalI
SalI
PstI
SmaI
BamHI
EcoRI
SalI
CAT-CmR-ccdB
attR2
EcoRV
P35SR2
T35S
RB
PstI
HindIII

E

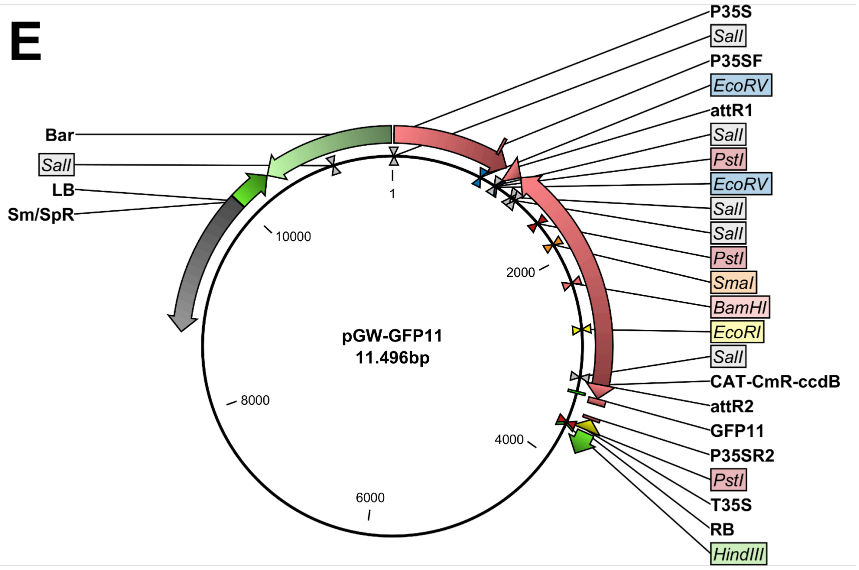
pGW-GFP11
11.496bp
1
2000
4000
6000
8000
10000
Bar
SalI
LB
Sm/SpR
P35S
SalI
P35SF
EcoRV
attR1
SalI
PstI
EcoRV
SalI
SalI
PstI
SmaI
BamHI
EcoRI
SalI
CAT-CmR-ccdB
attR2
GFP11
P35SR2
PstI
T35S
RB
HindIII
