# Supplementary material for: A Split-GFP Gateway Cloning System for Topology Analyses of Membrane Proteins in Plants
Source: PLoS One. 2017 Jan 13;12(1):e0170118. doi: 10.1371/journal.pone.0170118 (PMC5234810; doi:10.1371/journal.pone.0170118)
Supplement: S1 Table — (PDF) [file pone.0170118.s002.pdf]

A Split-GFP Gateway Cloning System for Topology Analyses of Membrane Proteins in Plants

Wenjun Xie, Mads Eggert Nielsen, Carsten Pedersen and Hans Thordal-Christensen

Plant Defence Genetics, Department of Plant and Environmental Sciences, University of Copenhagen, Thorvaldsensvej 40, DK-1871 Frederiksberg C, Denmark

S1 Table. Primers used in this work.

GFP11-M3-3F	CCGCACTAGTGATATCATGCGTGACCACATGGTCCTTC ATGAGTACGTAAATGCTGCTGGGATTACAACAAGTTT GTACAAAAAAGCAGGCTCCGCGGCCGCC
P35SR2	ATGAGCGAAACCCTATAAGAACC
GFP11-M3-4R	CATGGCCGCGGATATTTATGTAATCCCAGCAGCATTT ACGTACTCATGAAGGACCATGTGGTCACGCACCACTTT GTACAAGAAAGCTGGGTCGGCGCGCCCACCCTTGTAT GTTACTTGTTG
P35SF	ACAATCCCACTATCCTTCGCA
CNX-F	CACCATGGAACTGATTGAGAAAGCC
CNX-R	CTAATTATCACGTCTCGGTTGCC
CNX-R-noSTOP	ATTATCACGTCTCGGTTGCC
attB1-PEN1-F	AAAAAGCAGGCTACATGAACGATTTGTTTTCC
attB2-PEN1-R	AGAAAGCTGGGTCTCAACGCAATAGACGCCTTGC
attB1-F	GGGGACAAGTTTGTACAAAAAAGCAGGCTAC
attB2-R	GGGGACCACTTTGTACAAGAAAGCTGGGTC
PEN1mutSTOP-F	CAAGGCGTCTATTGCGTTTAGACCCAGCTTTCTTG
PEN1mutSTOP-R	CAAGAAAGCTGGGTCTAAACGCAATAGACGCCTTG
